# RNAi Screen and Proteomics Reveal NXF1 as a Novel Regulator of IRF5 Signaling

**DOI:** 10.1038/s41598-017-02857-z

**Published:** 2017-06-02

**Authors:** Bishi Fu, Mengmeng Zhao, Lingyan Wang, Girish Patil, Jennifer A. Smith, Ignacio J. Juncadella, Ljiljana Zuvela-Jelaska, Martin E. Dorf, Shitao Li

**Affiliations:** 1 Department of Microbiology & Immunobiology, Harvard Medical School, Boston, Massachusetts 02115 USA; 2 College of Life Science, Yangtze University, Jingzhou, Hubei 434025 People’s Republic of China; 3 Department of Physiological Sciences, Oklahoma State University, Stillwater, Oklahoma 74078 USA; 4 ICCB-Longwood Screening Facility, Harvard Medical School, Boston, Massachusetts 02115 USA; 5 Department of Immunology and Respiratory Diseases Research, Boehringer Ingelheim Pharmaceuticals Inc., Ridgefield, Connecticut 06877 USA

## Abstract

Interferon regulatory factor 5 (IRF5) is a key transcription factor of innate immunity, which plays an important role in host restriction to viral infection and inflammation. Genome-wide association studies have implied the association of IRF5 with several autoimmune diseases, including systemic lupus erythematosus (SLE), Sjogren’s syndrome, inflammatory bowel disease and multiple sclerosis. However, the regulation of IRF5-mediated immunity is not well understood. To uncover new regulators in IRF5 pathway, we used two “omics” approaches: affinity purification coupled with mass spectrometry and a high throughput RNAi screen. Proteomics identified 16 new IRF5 interactors while RNAi-mediated knockdown found 43 regulators of the TLR7-dependent IRF5 signaling pathway. NXF1 was identified in both screens. Stimulation with TLR7 ligand enhances formation of IRF5-NXF1 protein complexes. Gain or loss-of-function experiments revealed NXF1 selectively regulates TLR7-driven IRF5 transcriptional activity, suggesting a new role for NXF1 in the IRF5 signaling pathway.

## Introduction

IRF5 is a member of the interferon regulatory factor family of transcription factors driving the expression of type I interferons (IFN). In human immune cells, recognition of cognate Toll-like receptor 7 and 9 (TLR7 and TLR9) ligands leads to the activation of IRF5^1^, via the adaptor protein myeloid differentiation primary response gene 88 (MyD88). MyD88 recruits interleukin-1 receptor associated kinases (IRAKs) and tumor necrosis factor receptor associated factor 6 (TRAF6)^2, 3^. TANK binding kinase 1 (TBK1) and IκB kinase beta (IKKβ) phosphorylation of IRF5 leads to IRF5 dimerization and subsequent nuclear translocation^4–6^. Like other IRF family members, IRF5 has a prototypical helix-loop-helix and a conserved tryptophan repeat in its N-terminal DNA-binding domain. IRF5 induces expression of IFN and other cytokine genes by binding to promoters containing the IFN-stimulated response element (ISRE).

Although innate immunity is the front line of host defense against pathogens, an excessive innate immune response can cause autoimmune diseases. Recently, several genetic studies found an association between SLE and the various single nucleotide polymorphisms and functional variants of IRF5 gene. Other autoimmune diseases such as rheumatoid arthritis, Sjogren’s syndrome, systemic sclerosis, multiple sclerosis, and inflammatory bowel disease have also been associated with IRF5 polymorphisms, suggesting a role of IRF5 in common autoimmune disease pathways^7–10^. However, the regulatory mechanisms by which IRF5 contributes to autoimmune disease pathogenesis are still unclear^7–9^. To uncover the new regulators for IRF5-mediated innate immunity, we first used a proteomics approach to identify IRF5-interacting molecules. We also initiated a high throughput siRNA screen to define proteins which modulated IRF5 activity. Identification of new factors will advance the understanding of IRF5-mediated innate immunity.

## Results

### IRF5 isoforms demonstrate distinct activities on IFN induction

**Figure 1:**
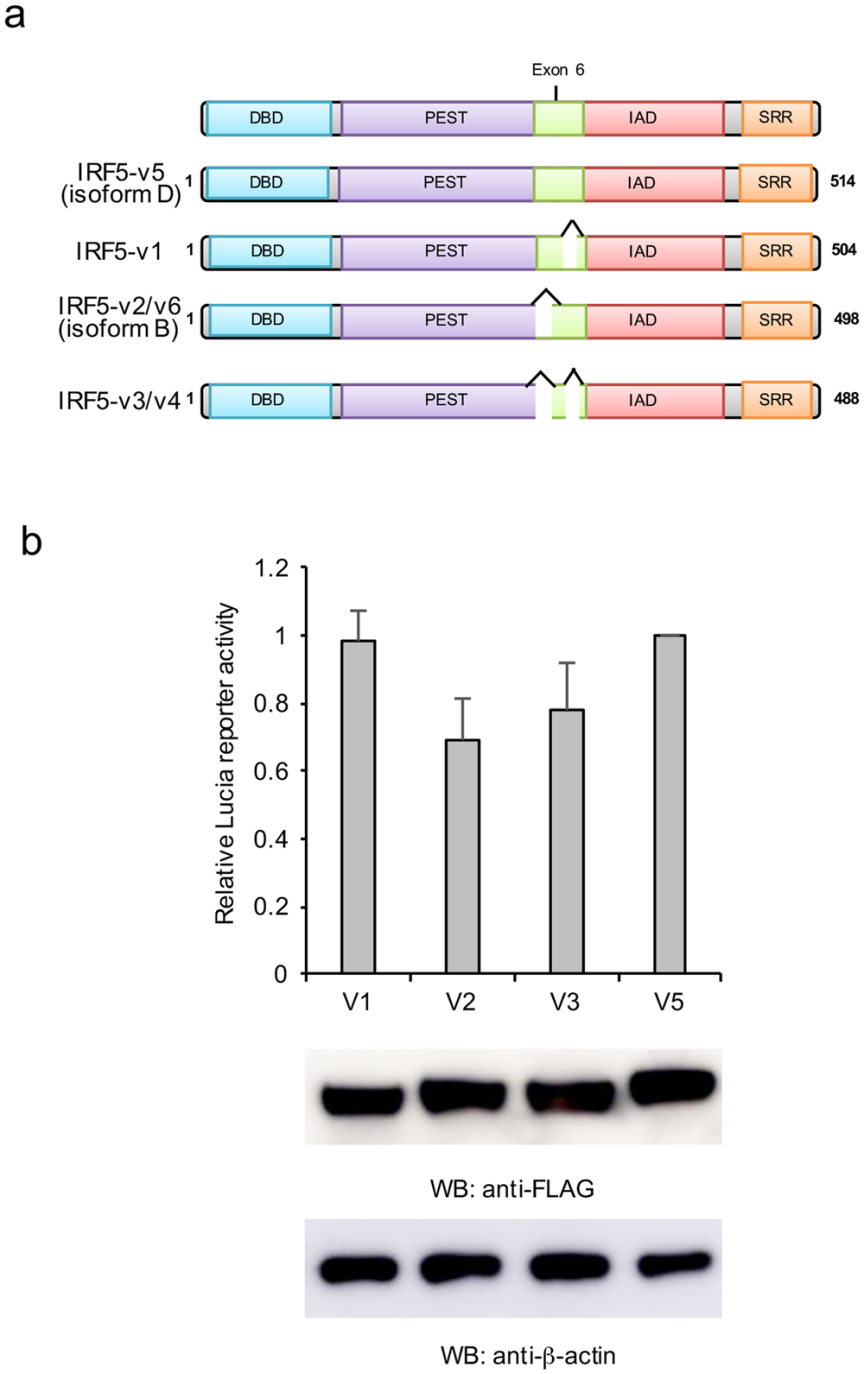
IRF5 variants activate IFN reporter. **(a**) A schematic of different IRF5 isoforms. DBD, DNA binding domain; PEST, region rich in proline (P), glutamic acid (E), serine (S) and threonine (T) residues; IAD, IRF association domain; SRR, Serine-Rich Region. “ ^” represents deleted regions. (**b**) IRF5 isoforms or mutants were transfected with Lucia reporter and TLR7 into HEK293 cells. After 48 hr, cells were mock treated or stimulated with 10 μg/ml R848 for 16 hr. Then cells were lysed and luciferase activities were examined. All experiments were repeated three times and two-tailed student’s *t*-test was performed. The protein expression levels were determined by Western Blot.

IRF5 splicing is a complex process resulting in at least a dozen IRF5 transcripts that can be differentially expressed in various cell types^11^. IRF5 variant 1 (v1), v2, v3, v4, v5 and v6 are expressed in immune cells, such as plasmacytoid dendritic cells, macrophages, and peripheral blood mononuclear cells^11^. Furthermore, IRF5-v5 encodes the longest protein isoform D whereas v1 has a 30-base pair in-frame indel in exon 6 (Fig. 1a). IRF5-v2 and -v6 use different promoters but encode the same protein isoform B that is 18 amino acids shorter than isoform D. IRF5-v3 and -v4 proteins have deletions in v1 and v2/v6^12^. However, the effects of these protein isoforms on IRF5 activity are not well known. Therefore, we investigated the activities of the predominant IRF5 isoforms using an IFN reporter assay. All these IRF5 isoforms were transfected with an IFN reporter, pNifty3-I-Lucia (Lucia) to the HEK293 cells stably expressing TLR7 (HEK293/TLR7). After 48 hr, cells were stimulated with 10 μg/ml R848, a TLR7 ligand. As shown in Fig. 1b, IRF5 transcript v1 and v5 induced the highest reporter activity whereas the other two IRF5 isoforms displayed lower activity. We chose IRF5 v5 (isoform D) for further study by proteomics and RNAi screening, as this isoform is highly expressed in primary peripheral blood mononuclear cells and encodes the longest peptide. We refer to isoform D as IRF5 throughout the paper, unless otherwise stated.

### Proteomics defines IRF5 protein interaction network

**Figure 2:**
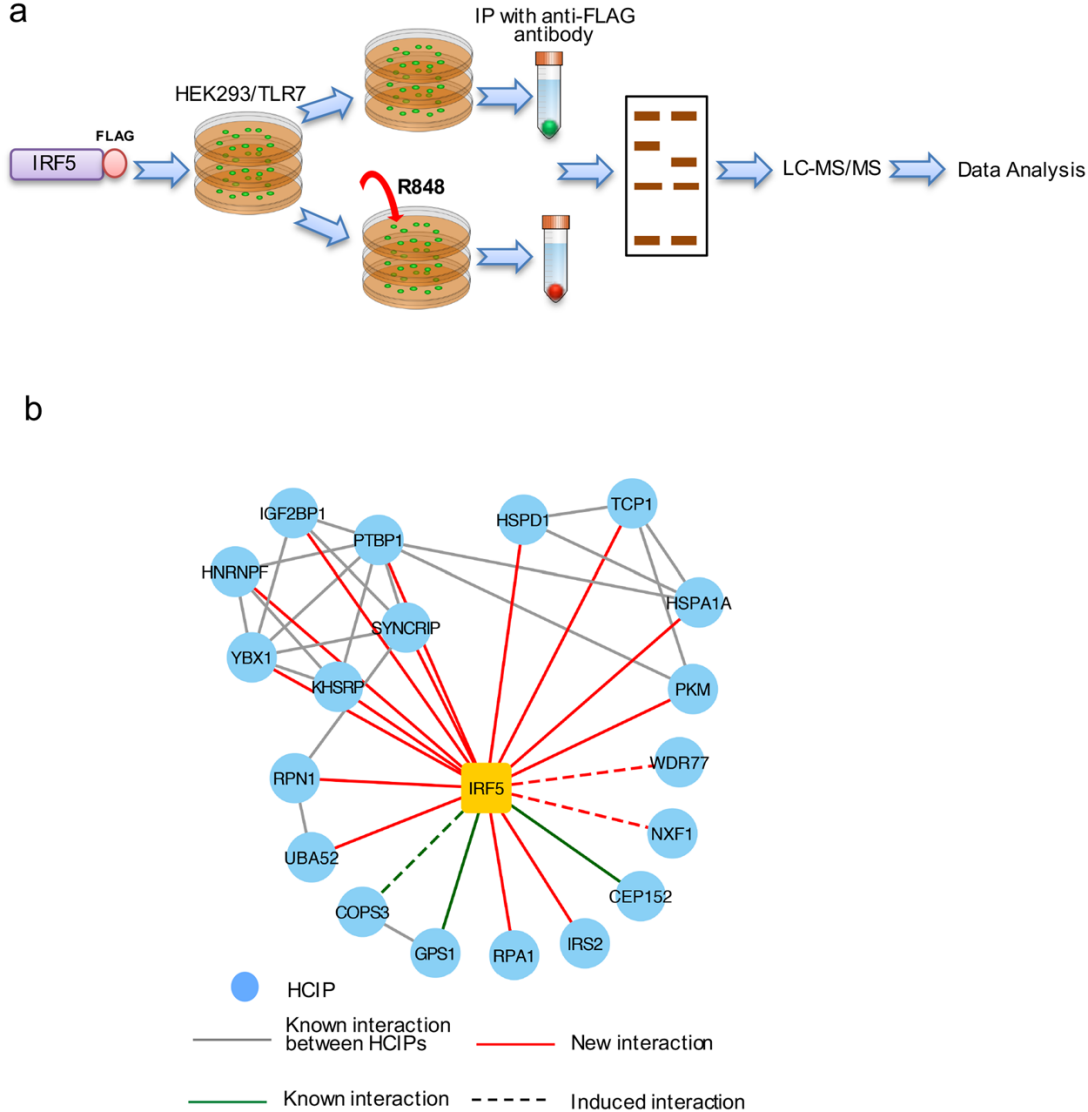
Affinity purification coupled with mass spectrometry (AP-MS) analysis of IRF5 protein complex. (**a**) Pipeline for AP-MS analysis of IRF5. (**b**) IRF5 protein interaction network. Legends are indicated. HCIP stands for high confidence candidate interacting protein.

Affinity purification coupled with mass spectrometry (AP-MS) for IRF5 is depicted in Fig. 2a. Briefly, FLAG-tagged IRF5 was transfected into HEK293/TLR7 cells and selected with hygromycin B for 14 days. Single cell clones were picked and expanded in 6-well plates. Protein expression levels in each clone were determined by immunoblotting. Cells from these stable cell lines were cultured in ten 15-cm plates. Half of the plates were stimulated with 10 μg/ml R848 for 4 hr. Cell lysates were immunoprecipitated with anti-FLAG antibody and eluted with 3X FLAG peptide. Samples were run on the NuPAGE gel and bands were excised for mass spectrometry.

The AP-MS for IRF5 was performed in biological duplicate. To efficiently reduce false positives in AP-MS, we adopted the statistical method SAINT^13^ in combination with large in-house proteomic database derived from HEK293 cells (161 protein complexes). Using a stringent statistical SAINT score (≥0.89), we identified 19 IRF5-interacting proteins (Fig. 2b and Supplementary Data 1). The results are consistent with the known interaction between COPS3, CEP152 and GPS1 with IRF5 isoform B^14^. In addition, it provides the evidence for novel 16 interactions. Three interactions (NXF1, WDR77, COPS3) are enhanced following stimulation with R848 ligand (Fig. 2b and Supplementary Data 1).

### Establishment of a stable IRF5 reporter cell line for RNAi screen

**Figure 3:**
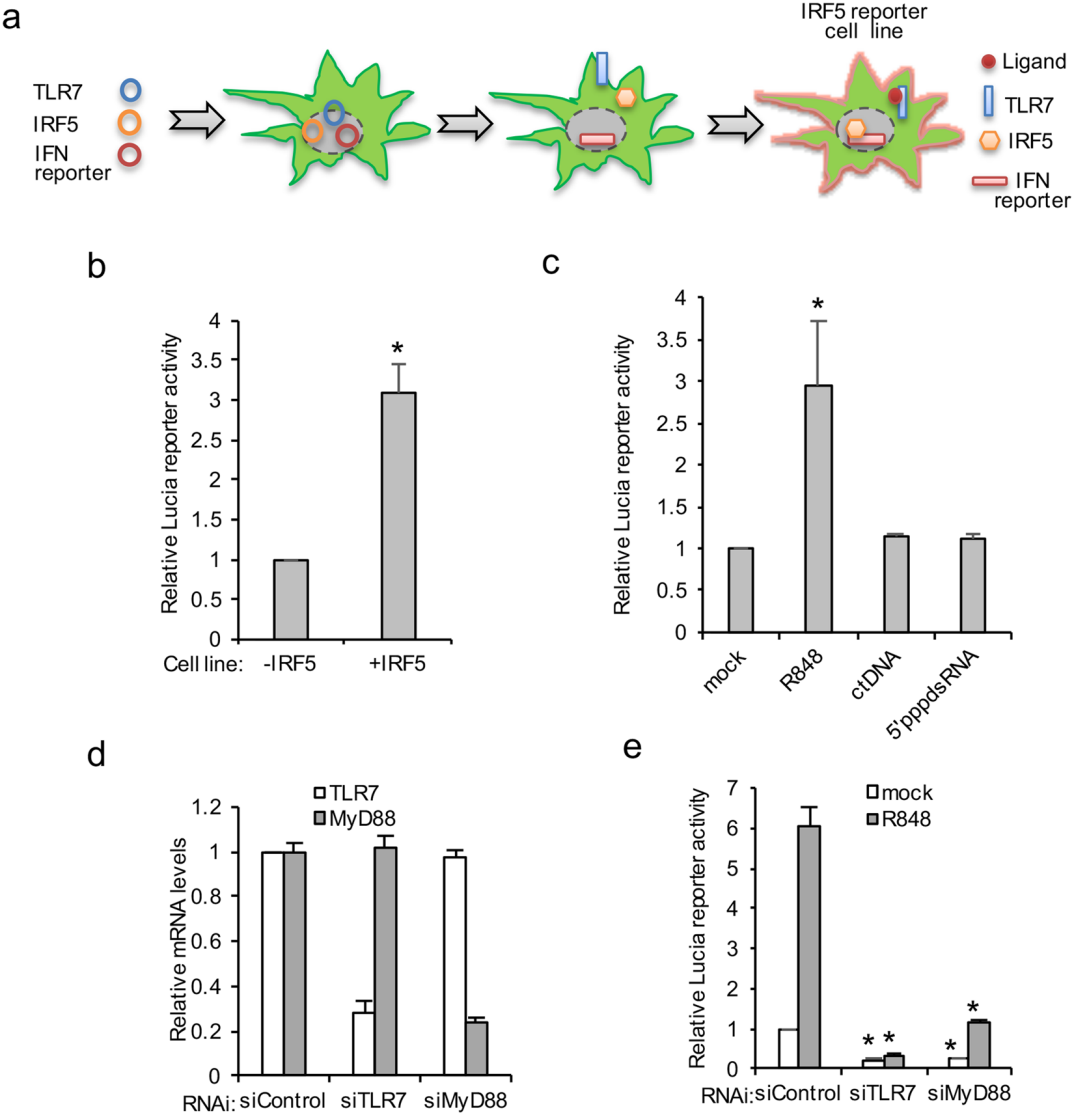
Characterization of IRF5 reporter cell line. (**a**) Illustration of procedures for generation of IRF5 reporter cell line. (**b**) HEK293/TLR7/Lucia cells expressing vector or IRF5 were stimulated with 10 μg/ml R848. After 16 hr, luciferase activity was measured. All experiments were repeated three times and two-tailed student *t*-test was performed. An asterisk indicates *p* < 0.05. (**c**) The IRF5 reporter cells were treated with 10 μg/ml R848, 1 μg/ml ctDNA or 1 μg/ml 5′-ppp-dsRNA. After 16 hr, luciferase activity was determined. (**d**) The IRF5 reporter cells were transfected with control siRNA, siTLR7 or siMyD88. After 72 hr, cells were treated with 10 μg/ml R848 for 16 hr. Cells were then collected and RNA was extracted. RT-PCR was performed to determine the relative levels of TLR7 and MyD88 mRNA expression which were normalized using the GAPDH housekeeping gene. (**e**) The IRF5 reporter cells were transfected with control siRNA or siRNA against TLR7 (siTLR7) or MyD88 (siMyD88). After 72 hr, cells were treated with 10 μg/ml R848 for 16 hr. Luciferase activity and CellTiter-Glo were measured. The ratio of luciferase to CellTiter-Glo was calculated. Relative activity was normalized by the control. All experiments were repeated three times and two-tailed student *t*-test was performed. An asterisk indicates *p* < 0.05.

Due to their high transfection efficiency, HEK293 cells were utilized as a model cell system to perform an IRF5-mediated high throughput genomic screen. We established an IRF5-specific reporter cell line by introducing IRF5 and the IFN Lucia reporter into HEK293/TLR7 cells (Fig. 3a). Toward this end, we first stably transfected the Lucia reporter into HEK293/TLR7 cells to establish the HEK293/TLR7/Lucia cell line. Next, we determined the requirement of IRF5 in response to the TLR7 ligand, R848 in HEK293/TLR7/Lucia cells. Empty vector or IRF5 was transfected into HEK293/TLR7/Lucia cells followed by stimulation with R848. R848 stimulation induced about 3.3-fold Lucia reporter activity in presence of IRF5 vs. vector (Fig. 3b), validating the requirement of IRF5 for optimal TLR7 signaling. Therefore, we further stably transfected IRF5 into HEK293/TLR7/Lucia cells to establish the HEK293/TLR7/Lucia/IRF5 cell line which is referred as IRF5 reporter cell line. Next, we examined the specificity of the IRF5 reporter cells to TLR7 ligand. The reporter cells were stimulated with different ligands of IFN activation, including R848, calf thymus DNA (ctDNA) and the viral RNA mimics, 5′ppp-dsRNA. As shown in Fig. 3c, R848 stimulation increased Lucia reporter activity while ctDNA and 5′ppp-dsRNA failed, suggesting the specificity of the IRF5 reporter cell line for R848 ligand. To substantiate that the IRF5 reporter cell line is a feasible tool for RNAi screening, we examined the effects of RNAi-mediated knockdown of TLR7 and MyD88, two essential genes in the TLR7-IRF5 pathway. The knock down efficiencies of each gene were ~70% (Fig. 3d). Knockdown of MyD88 or TLR7 reduced the basal and R848-induced reporter activity more than 3 fold (Fig. 3e). Taken together, these data demonstrate that we established an IRF5-specific reporter cell line that is amenable to siRNA-mediated knock down and responsive to TLR7 stimulation.

### siRNA screen on IRF5-mediated innate immunity

**Figure 4:**
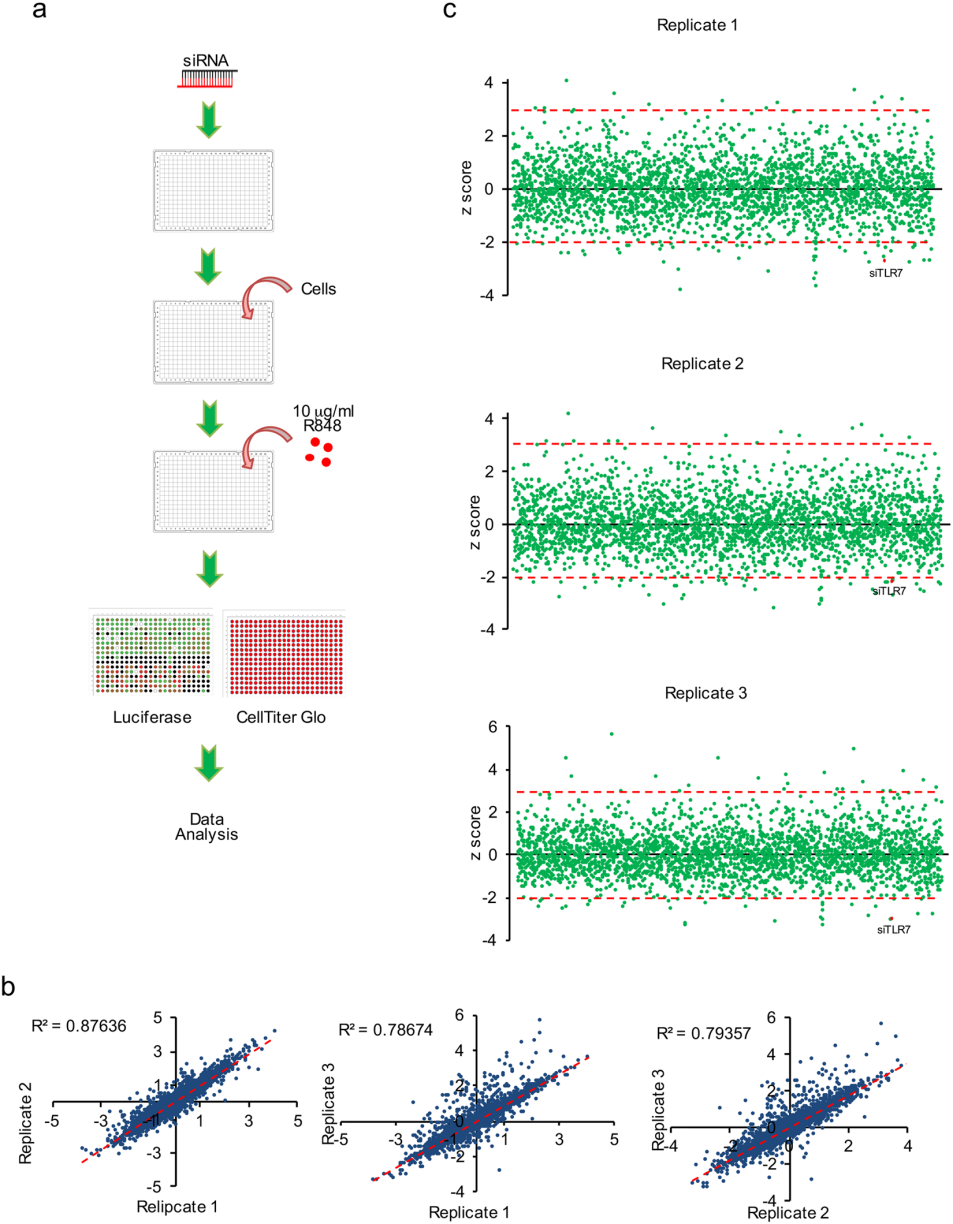
SMARTpool siRNA screen of IRF5 signaling pathway. **(a**) Outline of the screening procedures. (**b**) Determination of the R^2^ value of 3 replicates. (**c**) Summary of 3 replicates of SMARTpool RNAi screens. Z scores of 3 and -2 (P < 0.05) are indicated by red dash lines. The positive control siTLR7 is indicated.

To discover novel druggable regulators of IRF5 signaling pathway, we used the Dharmacon Focused Druggable Library at Harvard Medical School’s ICCB-Longwood Screening Facility, which represents a collection of 2,754 SMARTpools targeting 2,754 genes in a one gene/well format. In addition, we also screened the 19 genes identified by AP-MS that comprise an IRF5 protein interaction network. The high throughput screen outline is shown in Fig. 4a and the screen was conducted in triplicate. Briefly, cells were transfected with siRNA for 72 hr and stimulated with 10 μg/ml R848 for an additional 16 hr. Luciferase activity and CellTiter-Glo (CTG) were measured as readouts for IRF5 transcriptional activity and cellular viability, respectively. In addition to the experimental wells, each plate contained library- (Dharmacon non-targeting siRNAs, siKIF11, siPLK1) and assay-specific (siTLR7 and Dharmacon non-targeting siRNA#1) controls. Z′ factors were calculated for each plate and plates with a Z′ factor >0.2 were analyzed. In addition, we calculated the R2 values between the 3 replicates. They were ≥ 0.79, indicating high reproducibility (Fig. 4b).

For identification of primary hits, two parameters were included: luciferase expression and the relative cell number. After excluding those SMARTpools that decreased cellular viability by 30%, the ratio of luciferase to CellTiter-Glo was calculated for each well and z-score analysis performed on a per plate basis. A SMARTpool was considered a hit if the z-score was ≤ −2 or ≥ 3 (Fig. 4c and Supplementary Data 2) in at least two of the three replicates. We identified 60 potential hits from the primary SMARTpool RNAi screen (Supplementary Data 2).

### Validation of the RNAi screen

**Figure 5:**
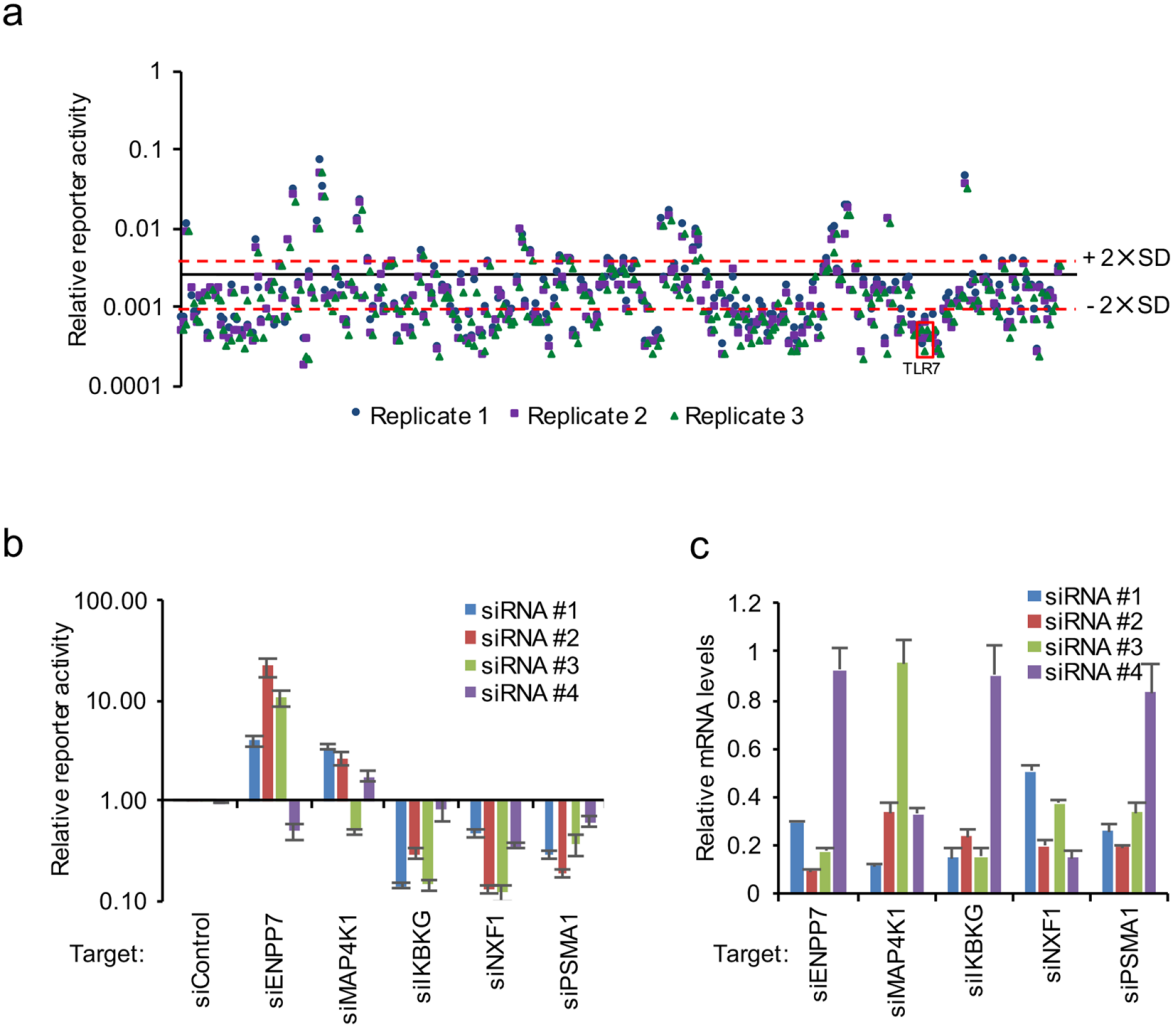
Validation of SMARTpool RNAi screen. (**a**) The hits from SMARTpool RNAi screen were selected for further validation by RNAi using individual siRNA duplexes. Four siRNA pairs per gene were individually transfected into the IRF5 reporter cells followed by treatment with 10 μg/ml R848 in three independent experiments. Luciferase activities and CellTiter-Glo were measured at 16 hr after R848 stimulation. ±2 × SD are indicated by red dash lines. The positive siTLR7 controls are boxed. (**b**) Four siRNA duplexes per gene were individually transfected into the IRF5 reporter cells followed by treatment with 10 μg/ml R848 in three independent experiments. Cell were collected at 16 h after R848 stimulation. Luciferase activities and CellTiter-Glo were measured. (**c**) Four siRNA duplexes per gene were individually transfected into the IRF5 reporter cells. After 72 hr, RNA was extracted for RT-PCR to determine the mRNA expression levels of each target gene.

To reconfirm the results from the primary screen, we next performed a deconvolution secondary screen in which the four siRNA duplexes comprising the hit SMARTpools were individually screened in a 1 duplex/well (4 wells/gene target) format. Additional assay-specific controls were included for data analysis. We confirmed 43 genes for which at least two siRNAs reduced or increased reporter activity by 60% (±2 × SD from mean of negative controls), without a concomitant reduction of cellular viability by >30% (Fig. 5a and Supplementary Data 3). We independently validated some of the cellular factors that were identified as repressors or inducers of IRF5 signaling. Thus, knockdown of ENPP7 or MAP4K1 increased R848-induced IFNβ mRNA expression (Fig. 5b). In contrast, RNAi knockdown of IKBKG, NXF1, or PSMA1 reduced R848-induced IFNβ mRNA levels (Fig. 5b). Functional activity correlated with knockdown efficiency of individual RNAi duplexes (Fig. 5c). Collectively, we validated 43 regulatory factors for IRF5 signaling pathway by secondary RNAi screen.

### Functional analysis reveals the role of proteasome in regulating the IRF5 pathway

**Figure 6:**
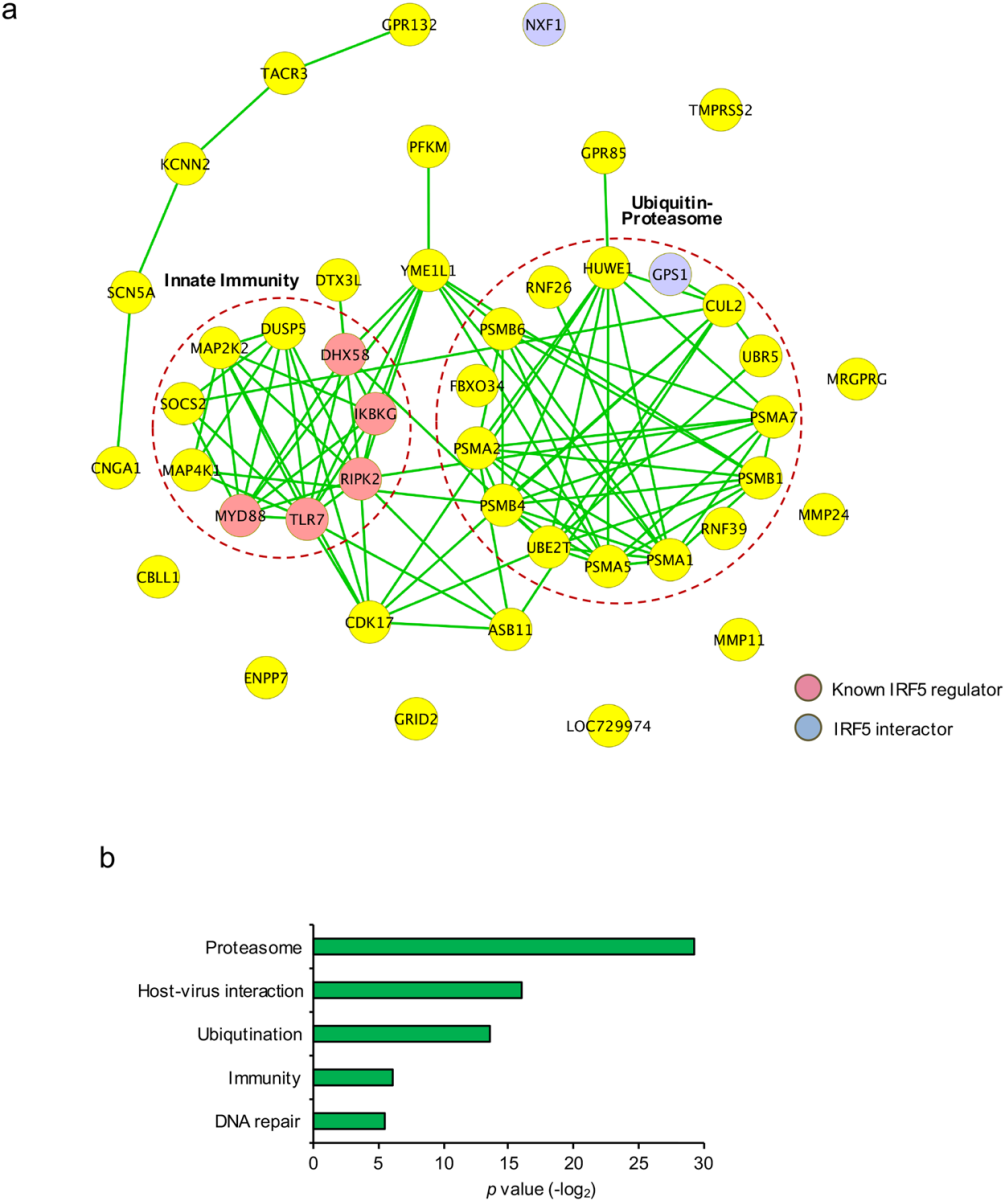
Functional analysis of IRF5 pathway. (**a**) STRING analysis of the validated RNAi hits. Medium confidence (0.4) of STRING was adopted. Pathways of innate immunity and proteasome are indicated by red dashed circle. (**b**) Negative log_2_ (*p* values) of enriched terms according to the GO of the cellular pathways.

We used STRING to derive an interaction network view of the 43 proteins considered hits after the secondary RNAi screen (Fig. 6a). Two of them were identified as IRF5 interactors by our proteomics analysis (NXF1 and GPS1) and five are known to regulate IRF5 signaling (MYD88, TLR7, RIPK2, IKBKG, DHX58)^3, 15, 16^. While not all proteins were connected via an interaction, two distinct “hubs” are visible: one involves innate immunity and another comprises 7 proteasome subunits and 8 proteins participating in ubiquitination. Similarly, Gene Ontology analysis identified several statistically enriched categories including innate immunity, ubiquitination and proteasome that were overrepresented among the 43 factors, underscoring the importance of these cellular functions in IRF5-mediated innate immunity (Fig. 6b).

### NXF1 regulates IRF5 signaling pathway

**Figure 7:**
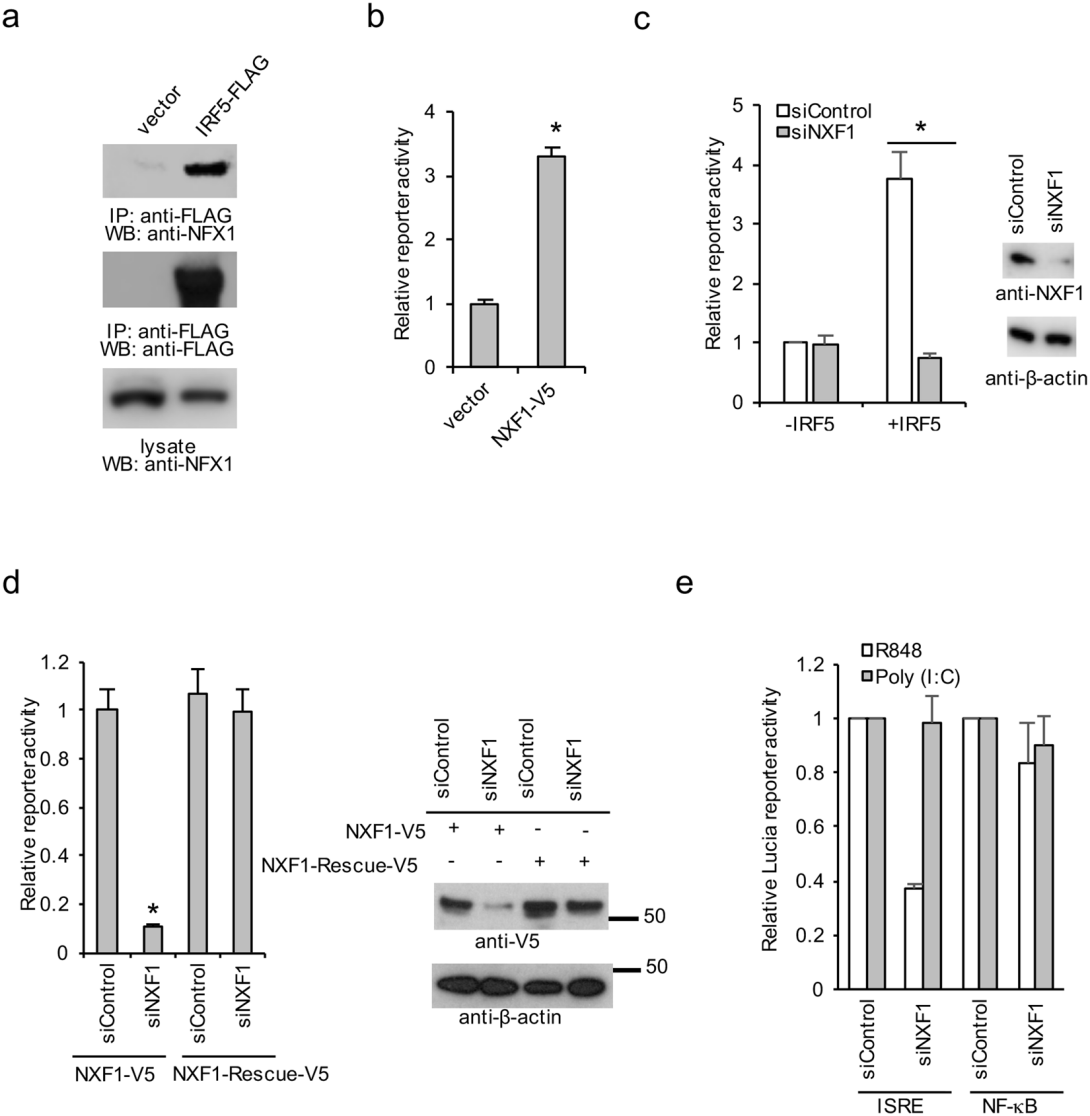
NXF1 regulates IRF5 pathway. (**a**) HEK293 cells were transfected with pCMV-3Tag-8 vector or IRF5-FLAG for 48 hr. Cell lysates were immunoprecipitated with anti-FLAG antibody and blotted with indicated antibodies. (**b**) NXF1-V5 or pCMV-3Tag8 vector was transfected into IRF5 reporter cells. After 48 hr, cells were stimulated with 10 μg/ml R848 for 16 hr. Then cells were lysed and luciferase activities were examined. All experiments were repeated three times and two-tailed student *t*-test was performed. (**c**) HEK293/TLR7/Lucia cells expressing vector or IRF5 were transfected with control siRNA or NXF1 siRNA. After 72 hr, cells were stimulated with 10 μg/ml R848 for 16 hr. Then cells were lysed and luciferase activities were examined. Western blot demonstrates the knockdown efficiency of endogenous NXF1. (**d**) IRF5 reporter cells were transfected with control siRNA or NXF1 siRNA together with wild type NXF1 or a rescue NXF1 cDNA which is resistant to the siRNA. The *P* value was calculated (two-tailed Student’s *t*-test) by comparison to the siRNA control. An asterisk indicates *P* < 0.05. Western blot demonstrates the knockdown efficiency. (**e**) THP-1 cells expressing a ISRE-luciferase and a NF-κB reporter were transfected with control siRNA or NXF1 siRNA pairs. After 72 hr, cells were incubated with 10 μg/ml R848 or 1 μg/ml poly (I:C) for 16 hr. Cells were lysed and SEAP (NF-κB) and luciferase (ISRE) activities were examined.

NXF1 was identified by both proteomics and RNAi screening (Fig. 6a). To verify the interaction between IRF5 and NXF1, IRF5-FLAG was transfected into HEK293 cells for 48 hr. Cell lysates were then immunoprecipitated with anti-FLAG antibody and blotted with anti-NXF1 antibody. Immunoprecipitation showed that IRF5-FLAG interacted with endogenous NXF1 (Fig. 7a).

We next overexpressed NXF1 in IRF5 reporter cells. Overexpression of NXF1 increased IRF5 reporter activity (Fig. 7b). RNAi depleted endogenous NXF1 protein and reduced IRF5 reporter activity in HEK293 cells (Fig. 7c). However, knockdown of NXF1 had little effect on control cells not transfected with IRF5 (Fig. 7c), suggesting a requirement of NXF1 for IRF5 signaling activity. To exclude off-target effects of RNAi, cells were transfected with a siRNA-resistant NXF1 rescue expression construct. The rescue construct restored IRF5 activity, validating siRNA specificity (Fig. 7d). To examine the specificity of NXF1 in a physiologic system reliant on endogenous TLR7 and endogenous IRF5, we chose the human monocytic THP-1 cell line. THP-1 cells expressing ISRE and NF-κB reporters were transfected with control siRNA or NXF1 siRNA duplexes. Cells were stimulated with the TLR7 ligand or the TLR3 ligand, poly (I:C) for 16 hr. As shown in Fig. 7e, knockdown of NFX1 selectively reduced ISRE activity induced by the TLR7 ligand, but not the TLR3 ligand. NFX1 had little effect on R848 or poly(I:C) induced NF-κB activity (Fig. 7e), supporting a specific role for NXF1 in the TLR7-IRF5-ISRE transcriptional pathway. Taken together, the data suggest NXF1 selectively regulates TLR7-dependent IRF5 signaling.

## Discussion

Although considerable knowledge exists regarding IRF5 function, the regulatory factors in IRF5-mediated innate immunity are less understood. Therefore, we executed two “omics” screens and identified 43 proteins capable of modulating the TLR7-IRF5 signaling pathway. As with any large screening effort, these candidates need additional validation, including examination of potential off-target effects and determination of any cell- or stimulus-specific effects. Regardless, these experiments demonstrated IRF5 regulators are enriched for genes involved in immunity and host-virus interaction, confirming the robustness of our assays.

We found that genes involved in innate immunity and the proteasome are two major “cores” in the IRF5 regulatory network. In fact, proteasome inhibitors can alleviate lupus in mice and recently have been applied for clinical study on refractory SLE^17, 18^. The rationale of using proteasome inhibitors for treatment of autoimmune diseases is due to their ability to (a) inhibit the activation of NF-κB and the production of cytokines, such as IFN; and (b) induce apoptosis of activated immune cells. Neubert *et al*.^19^ demonstrated that bortezomib, a proteasome inhibitor, depleted plasma cells, thereby ameliorating lupus symptoms and prolonging survival in 2 mouse strains. Recently, Ichikawa *et al*. showed that TLR-induced IFN production in mice was completely abrogated by proteasome inhibition^20^. The proteasome is the final processing factory for ubiquitinated protein. Interestingly, our proteomics identified two components (GPS1 and COPS3) of the COP9 signalosome complex (CSN). We confirmed GPS1 regulates IRF5 signaling in our subsequent siRNA screen. The CSN complex is an essential regulator of the ubiquitin conjugation pathway; it mediates the deneddylation of the cullin subunits of SCF-type E3 ligase complexes^21^. A previous study reported that CSN controlled NF-κB by deubiquitinylation of IκBα^22^. In addition, the CSN associates with IRF5 to promote its stability^23^. However, the mechanism by which ubiquitin-proteasome regulates IRF5 signaling is not clear at the current stage and will be investigated in the future.

This report identifies NXF1 as a positive regulator of the TLR7-IRF5 pathway. NXF1 is a ubiquitous RNA binding protein localized throughout the nucleus. NXF1 is an mRNA export receptor which binds processed mRNA and escorts it through nuclear pores^24^. Endogenous NXF1 was detected in IRF5 complexes and interactions between IRF5 and NXF1 are enhanced following activation with TLR7 ligand. Knockdown of NXF1 with siRNA selectively inhibits TLR7-dependent ISRE signaling. Overexpression further corroborates the role of NXF1 in the IRF5 signaling pathway. The combined findings support a novel role for NXF1 in the TLR7-IRF5-ISRE signaling pathway. Additional studies will be required to define the molecular mechanisms of NXF1 action on IRF5 transcriptional responses.

As this study focused primarily on the discovery of new regulators of IRF5, further investigation of the molecular mechanisms of each candidate will provide new insight into the IRF5 signaling pathway and provide new opportunities for the development of antiviral therapies and therapeutic targets for autoimmune diseases.

## Methods

### Cells

HEK293 cells (ATCC, Catalog #CRL-1573) were maintained in Dulbecco’s Minimal Essential Medium (Life Technologies, Catalog #11995-065) containing antibiotics (Life Technologies, Catalog #15140-122) and 10% Fetal Bovine Serum (Life Technologies, Catalog #26140-079). THP-1 Dual cells (Invivogen, Catalog #thpd-nfis) were maintained in RPMI 1640 (Life Technologies, Catalog #11875-085) containing antibiotics and 10% Fetal Bovine Serum.

### Antibodies and Reagents

Anti-β-actin (Abcam, Catalog #ab8227, WB (1:1000)), anti-FLAG (Sigma, Catalog #F3165, WB (1:1000)), anti-NXF1 (Cell Signaling, Catalog #12735, WB (1:1000)), anti-V5 (Thermo Fisher Scientific, Catalog #MA5-15253, WB (1:1000)), Goat anti-mouse IgG-HRP (Santa Cruz Biotechnology, Catalog #sc-2055, WB (1:10000)), Goat anti-rabbit IgG-HRP (Santa Cruz Biotechnology, Catalog #sc-2030, WB (1:10000)).

R848 (Catalog #tlrl-R848-5), and 5′ppp-dsRNA (Catalog #tlrl-3prna) were purchased from Invivogen (San Diego, CA). Calf thymus DNA was purchased from Thermo Fisher Scientific (Catalog #15633019).

### Plasmids

Human IRF5 v5 (NM_001098629 with codon optimization) and v2 (NM_032643) were cloned into pCMV-3Tag-8 (Agilent Technologies, Catalog #240203) and fused with FLAG epitope. FLAG tagged IRF5 v1 was made by deletion of 30 nt (571–600) of pCMV3Tag8-IRF5 v5 using QuikChange II Site-Directed Mutagenesis kit (Agilent Technologies, Catalog #200523). The IRF5 v5 encoding protein isoform D was cloned into pQCXIP (Clontech, Catalog #631516) to make pQCXIP-IRF5. C-terminal V5 tagged human NXF1 was provided by the Dana Farber/Harvard Cancer Center DNA Resource Core (Catalog #HsCD00418053).

pNifty3-I-Lucia (Invivogen, Catalog #pnf3-lc4) comprises the mouse interferon beta minimal promoter, five ISRE transcription factor binding sites and a secreted luciferase (Lucia) reporter gene. pCMV3Tag8-Lucia was made by cloning Lucia into the pCMV-3Tag-8 (Agilent Technologies).

### Real-time PCR

Total RNA was prepared using RNeasy columns (Qiagen, Catalog #74136). One µg RNA was transcribed into cDNA using QuantiTect reverse transcription kit (Qiagen, Catalog #205311). For one real-time reaction a 20 µl SYBR Green PCR reaction mix (Roche Applied Science) including 1/10 of the synthesized cDNA plus an appropriate oligonucleotide primer pair were run on the LightCycler 480 (Roche). The comparative Ct method was used to determine relative mRNA expression of genes as normalized by the housekeeping gene, GAPDH as detailed elsewhere^14^.

### Plasmid Transfection

Control vector or plasmids were transfected individually into HEK293 cells using Lipofectamine 2000 Transfection Reagent (Life Technologies, Catalog #11668-019) according to manufacturer’s protocol.

### Establishment of the IRF5 reporter cell line

HEK 293/TLR7 cell line (Invivogen, Catalog #293xl-htlr7) was transfected with pCMV3tag8-Lucia and selected with 100 µg/ml Hygromycin. After selection, the stable cell line (named HEK 293/TLR7/Lucia) was further transfected with pQCXIP-IRF5 and selected with 1 µg/ml Puromycin to make the IRF5 reported cell line. The IRF5 reporter cell line was maintained in medium supplemented with 100 µg/ml Normocin, 10 µg/ml Blasticidin, 100 µg/ml Hygromycin and 1 µg/ml Puromycin.

### Sample preparation, Western blot and Immunoprecipitation

1 × 10^6^ cells were lysed in 500 μl TAP lysis buffer (50 mM Tris-HCl [pH 7.5], 10 mM MgCl2, 100 mM NaCl, 0.5% Nonidet P40, 10% glycerol, Complete EDTA-free protease inhibitor cocktail tablets (Roche, Catalog #11873580001)) for 30 min at 4 °C. Then lysates are centrifuged for 10,000X rpm for 30 min. Supernatant was collected and mixed with 1X Lane Marker Reducing Sample Buffer (Thermo Fisher Scientific, Catalog #39000).

Precision Plus Protein Dual Color Standards (5 μl) (Bio-Rad, Catalog #161-0374) and samples (10–15 μl) were loaded into Mini-Protean TGX Precast Gel, 15-well (Bio-Rad, Catalog #456-103) and run in 1X Tris/Glycine/SDS Buffer (Bio-Rad, Catalog #161-0732) for 22 min at 200 volts. Protein samples were transferred to Immun-Blot PVDF Membrane (Bio-Rad, Catalog #162-0177) in 1X Tris/Glycine Buffer (Bio-Rad, Catalog #161-0734) at 70 V for 60 min. PVDF membrane were blocked in 1X TBS buffer (Bio-Rad, Catalog #170-6435) containing 5% nonfat milk, Blotting-Grade Blocker (Bio-Rad, Catalog #170-6404) for 1 hr. After washing with 1X TBS buffer for 30 min, the membrane blot was incubated with appropriately diluted primary antibody in antibody dilution buffer (1X TBS, 5% BSA, 0.05% sodium azide) at 4 °C for 16 hr. Then the blot was 3X washed with 1X TBS (each time for 10 min) and incubated with secondary HRP-conjugated antibody in antibody dilution buffer (1:10000 dilution) at room temperature for 1 hr. After 3 washes with 1X TBS (each time for 10 min), the blot was incubated with Clarity Western ECL Substrate (Bio-Rad, Catalog #170-5060) for 1–2 min. The membrane was removed from the substrates, then exposed either to HyBlot CL Autoradiography Film (Denville Scientific Inc. #E3018) in the dark room or to Amersham imager 600 (GE Healthcare Life Sciences, Marlborough, MA). Uncropped scans of western blots are provided in Supplementary Figure 1.

For immunoprecipitation, 2% of cell lysates (10^6^ cells) were saved for input control and the remainder was incubated with 10 μl EZview Red Anti-FLAG M2 Affinity Gel (Sigma, Catalog #F2426). After mixing end-over-end at 4 °C overnight, the beads were 3X washed (1 min for each wash) with 500 μl lysis buffer. Anti-FLAG M2 Affinity Gel was eluted with 0.5 mg/ml 3X FLAG peptide (Sigma, Catalog #F4799).

### RNAi Screen

For high-throughput screening, 384 well assay plates (Corning, Catalog #3570) were prepared with 8.5 μl/well Lipofectamine RNAiMAX/Opti-MEM mixture (0.14 μl RNAiMAX, 8.36 μl Opti-MEM) using the Thermo Multidrop Combi plate filler. 1.25 μl of 1 µM siRNA was then added to each 384-well assay plate using an Agilent Bravo Automated liquid handing system, followed by triturating (5 µl) three times per well. While the siRNA and transfection reagent complexed at room temperature, cells were trypsinized and pipetted into single-cell suspensions (5 × 10^4^/ml). 2000 cells in 40 μl of DMEM supplemented with 10% FBS were seeded on top of the siRNA-lipofectamine mixture. After centrifugation for 1 min at 1000X rpm, cells were incubated for 72 hr at 37 °C in 5% CO_2_. Afterward 10 μl culture medium containing 10 μg/ml R848 was added to each well. 16 hr later, 10 μl culture supernatant was transferred to new 384 plates (Corning #3570) containing 50 µl/well luciferase assay reagent (Invivogen, Catalog #rep-qlc). After 5 min the plates were read using a Perkin Elmer EnVision multi-mode plate reader to quantitate luciferase levels.

Cell number was assessed by using the CellTiter-Glo Luminescent Cell Viability Assay (Promega, Catalog #G7571) to quantitate ATP levels. Briefly, the assay plates were left at room temperature for 20 min, then 40 µl CellTiter-Glo reagent was added to each well using the Combi. Following a 10 min incubation, luminescence was quantitated using the Envision.

The Focused Druggable Library Human (Catalog #G-004675), control siGENOME non-targeting siRNA#3 (Catalog #D-001210-03-05) and siTLR7 (Catalog #M-004714-01) were obtained from Dharmacon.

### Screen Data Analysis

For each individual screening plate, Z′ factor is used as quality control. Z′ factor is a dimensionless calculation used to assess the quality of a population of sample compounds tested^25^. The Z′ value was calculated as follows: Z′ = 1 − (3 × SD of positive control + 3 × SD of negative control)/|mean of positive control – mean of negative control|, where SD represents the standard deviation. Z′ values more than 0.2 were considered acceptable for high throughput screening.

For screen data analysis, normalized values (N), where N = (Lucia luciferase value)/(CellTiter-Glo value), were calculated. We chose the log transformation analysis because the data fit in a linear progression for both increases and decreases with respect to the plate average^26, 27^. For statistical analysis the data, Z-scores were calculated. Genes with Z score ≥2 or ≤−2 were considered potential hits.

### Purification of IRF5 protein complex and mass spectrometry

Affinity purification coupled with mass spectrometry (AP-MS) experiments were performed as previously described^14^. For protein purification, HEK293 cell lines stably expressing IRF5-FLAG divided into 2 groups. Each group of cells was expanded and cultured in five 15-cm dishes. Then, one group was treated with 1 μg/ml R848. After 16 hr, the cells were collected and lysed in 10 ml of TAP buffer (50 mM Tris-HCl [pH 7.5], 10 mM MgCl_2_, 100 mM NaCl, 0.5% Nonidet P40, 10% glycerol, phosphatase inhibitors and protease inhibitors)^28^. Cell lysates were precleared with 50 μl of protein A/G resin before the addition of 20 μl of anti-FLAG resin and incubation for 16 hr at 4 °C on a rotator. The resin was washed 3 times and transferred to a spin column (Sigma) with 40 μl of 3X FLAG peptide for 1 hr at 4 °C on a rotator. The purified complexes were loaded onto a 4–12% NuPAGE gel (Invitrogen, Catalog #NP0323BOX). The gels were stained with a SilverQuest staining kit (Invitrogen, Catalog #LC6070), and lanes were excised for mass spectrometry analysis by the Taplin Biological Mass Spectrometry Facility (Harvard Medical School, Boston, MA).

### SAINT analysis of AP-MS data

Two independent purifications of IRF5-FLAG were analyzed by AP-MS. The resulting data are presented in Supplementary Data 1, which were compared with our database of 161 samples from stable HEK293 cell lines expressing FLAG tag-fused proteins handled in identical fashion. The SAINT algorithm (http://sourceforge.net/projects/saint-apms) was used to evaluate the MS data^13^. The default SAINT options were low Mode = 1, min Fold = 0, and norm = 0. The SAINT scores computed for each biological replicate were averaged (AvgP) and reported as the final SAINT score. The fold change was calculated for each prey protein as the ratio of spectral counts from replicate bait purifications to the spectral counts across all negative controls. A background factor of 0.1 was added to the average spectral counts of negative controls to prevent division by zero. The proteins included in the final interactome list had an AvgP ≥0.89. The threshold for SAINT scores was selected based on receiver operating curve analysis performed using publicly available protein interaction data and the FLAG AP-MS data set as a list of true positive interactions. A SAINT score of AvgP ≥0.89 was considered a true positive BioID protein with an estimated FDR of ≤2%.

### Bioinformatic Analysis

The IRF5 protein interaction network was generated in Cytoscape (www.cytoscape.org). The interactions of IRF5 regulators were analyzed in STRING (http://string-db.org) with the confidence score of 0.4. Gene Ontology analysis was performed using DAVID 6.8 Beta (https://david-d.ncifcrf.gov).

## Electronic supplementary material

Supplementary Figure

Supplementary Dataset 1

Supplementary Dataset 2

Supplementary Dataset 3

## References

[1] Takeuchi O, Akira S (2010). Pattern recognition receptors and inflammation. Cell.

[2] Takaoka A (2005). Integral role of IRF-5 in the gene induction programme activated by Toll-like receptors. Nature.

[3] Balkhi MY, Fitzgerald KA, Pitha PM (2008). Functional regulation of MyD88-activated interferon regulatory factor 5 by K63-linked polyubiquitination. Mol Cell Biol.

[4] Lopez-Pelaez M (2014). Protein kinase IKK beta-catalyzed phosphorylation of IRF5 at Ser462 induces its dimerization and nuclear translocation in myeloid cells. P Natl Acad Sci USA.

[5] Ren JY, Chen X, Chen ZJJ (2014). IKK beta is an IRF5 kinase that instigates inflammation. P Natl Acad Sci USA.

[6] Cheng TF (2006). Differential activation of IFN regulatory factor (IRF)-3 and IRF-5 transcription factors during viral infection. J Immunol.

[7] Cham CM, Ko K, Niewold TB (2012). Interferon regulatory factor 5 in the pathogenesis of systemic lupus erythematosus. Clin Dev Immunol.

[8] Eames HL, Corbin AL, Udalova IA (2016). Interferon regulatory factor 5 in human autoimmunity and murine models of autoimmune disease. Transl Res.

[9] Lazzari E, Jefferies CA (2014). IRF5-mediated signaling and implications for SLE. Clin Immunol.

[10] Stone RC (2012). Interferon regulatory factor 5 activation in monocytes of systemic lupus erythematosus patients is triggered by circulating autoantigens independent of type I interferons. Arthritis Rheum-Us.

[11] Graham RR (2006). A common haplotype of interferon regulatory factor 5 (IRF5) regulates splicing and expression and is associated with increased risk of systemic lupus erythematosus. Nat Genet.

[12] Mancl ME (2005). Two discrete promoters regulate the alternatively spliced human interferon regulatory factor-5 isoforms. J Biol Chem.

[13] Choi H (2011). SAINT: probabilistic scoring of affinity purification-mass spectrometry data. Nat Methods.

[14] Li S, Wang L, Berman M, Kong YY, Dorf ME (2011). Mapping a dynamic innate immunity protein interaction network regulating type I interferon production. Immunity.

[15] Pandey AK (2009). NOD2, RIP2 and IRF5 play a critical role in the type I interferon response to Mycobacterium tuberculosis. PLoS Pathog.

[16] Schoenemeyer A (2005). The interferon regulatory factor, IRF5, is a central mediator of toll-like receptor 7 signaling. J Biol Chem.

[17] Alexander T (2015). The proteasome inhibitior bortezomib depletes plasma cells and ameliorates clinical manifestations of refractory systemic lupus erythematosus. Ann Rheum Dis.

[18] Verbrugge, S. E., Scheper, R. J., Lems, W. F., de Gruijl, T. D. & Jansen, G. Proteasome inhibitors as experimental therapeutics of autoimmune diseases. *Arthritis Res Ther* **17**, doi:ARTN 1710.1186/s13075-015-0529-1 (2015). 10.1186/s13075-015-0529-1 PMC4308859 25889583

[19] Neubert K (2008). The proteasome inhibitor bortezomib depletes plasma cells and protects mice with lupus-like disease from nephritis. Nat Med.

[20] Ichikawa HT (2012). Beneficial effect of novel proteasome inhibitors in murine lupus via dual inhibition of type I interferon and autoantibody-secreting cells. Arthritis Rheum.

[21] Wei N, Serino G, Deng XW (2008). The COP9 signalosome: more than a protease. Trends Biochem Sci.

[22] Schweitzer K, Bozko PM, Dubiel W, Naumann M (2007). CSN controls NF-kappaB by deubiquitinylation of IkappaBalpha. EMBO J.

[23] Korczeniewska J, Barnes BJ (2013). The COP9 signalosome interacts with and regulates interferon regulatory factor 5 protein stability. Mol Cell Biol.

[24] Natalizio BJ, Wente SR (2013). Postage for the messenger: designating routes for nuclear mRNA export. Trends Cell Biol.

[25] Zhang JH, Chung TD, Oldenburg KR (1999). A Simple Statistical Parameter for Use in Evaluation and Validation of High Throughput Screening Assays. J Biomol Screen.

[26] DasGupta R, Kaykas A, Moon RT, Perrimon N (2005). Functional genomic analysis of the Wnt-wingless signaling pathway. Science.

[27] Crow YJ (2006). Mutations in the gene encoding the 3′-5′ DNA exonuclease TREX1 cause Aicardi-Goutieres syndrome at the AGS1 locus. Nature genetics.

[28] Fu B (2015). TRIM32 Senses and Restricts Influenza A Virus by Ubiquitination of PB1 Polymerase. PLoS pathogens.

