# Supplementary material for: RNAi Screen and Proteomics Reveal NXF1 as a Novel Regulator of IRF5 Signaling
Source: Sci Rep. 2017 Jun 2;7:2683. doi: 10.1038/s41598-017-02857-z (PMC5457443; doi:10.1038/s41598-017-02857-z)
Supplement: Supplementary file 1 — Supplementary Figure [file 41598_2017_2857_MOESM1_ESM.pdf]

RNAi Screen and Proteomics Reveal NXF1 as a Novel Regulator of IRF5 Signaling

Bishi Fu, Mengmeng Zhao, Lingyan Wang, Girish Patil, Jennifer A. Smith, Ignacio J. Juncadella, Ljiljana Zuvela-Jelaska, Martin E. Dorf, Shitao Li

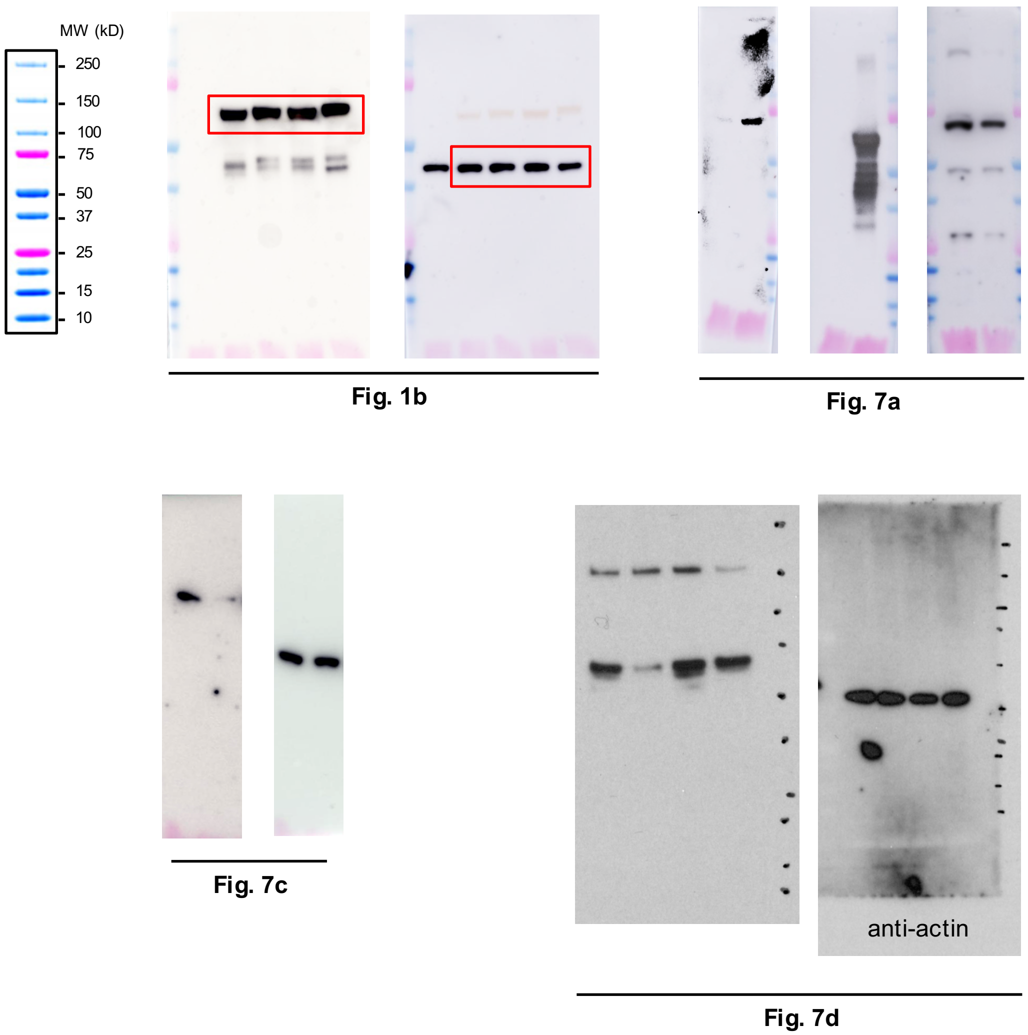

Supplementary Figure 1. Uncropped immunoblots . Labeling below each panel refers to the corresponding figure in the article. Red frame indicates the bands of the protein of interest.
